# Antidepressant drug prescription and incidence of COVID-19 in mental health outpatients: a retrospective cohort study

**DOI:** 10.1186/s12916-023-02877-9

**Published:** 2023-06-21

**Authors:** Oleg O. Glebov, Christoph Mueller, Robert Stewart, Dag Aarsland, Gayan Perera

**Affiliations:** 1 Institute of Neuroregeneration and Neurorehabilitation, Department of Pathophysiology, School of Basic Medicine, Qingdao University, Shandong, China; 2 Institute of Psychiatry, Psychology and Neuroscience, King’s College London, London, UK; 3 South London and Maudsley National Health Service Foundation Trust, London, UK; 4 Centre for Age-Related Research, Stavanger University Hospital, Stavanger, Norway

**Keywords:** COVID-19, SSRI, Antidepressants, Drug repurposing, Respiratory infection, SARS-CoV-2

## Abstract

**Background:**

Currently, the main pharmaceutical intervention for COVID-19 is vaccination. While antidepressant (AD) drugs have shown some efficacy in treatment of symptomatic COVID-19, their preventative potential remains largely unexplored. Analysis of association between prescription of ADs and COVID-19 incidence in the population would be beneficial for assessing the utility of ADs in COVID-19 prevention.

**Methods:**

Retrospective study of association between AD prescription and COVID-19 diagnosis was performed in a cohort of community-dwelling adult mental health outpatients during the 1st wave of COVID-19 pandemic in the UK. Clinical record interactive search (CRIS) was performed for mentions of ADs within 3 months preceding admission to inpatient care of the South London and Maudsley (SLaM) NHS Foundation Trust. Incidence of positive COVID-19 tests upon admission and during inpatient treatment was the primary outcome measure.

**Results:**

AD mention was associated with approximately 40% lower incidence of positive COVID-19 test results when adjusted for socioeconomic parameters and physical health. This association was also observed for prescription of ADs of the selective serotonin reuptake inhibitor (SSRI) class.

**Conclusions:**

This preliminary study suggests that ADs, and SSRIs in particular, may be of benefit for preventing COVID-19 infection spread in the community. The key limitations of the study are its retrospective nature and the focus on a mental health patient cohort. A more definitive assessment of AD and SSRI preventative potential warrants prospective studies in the wider demographic.

## Background

More than 3 years since the declaration of the global pandemic, COVID-19 remains a major public health concern across the world. In the beginning of the pandemic, the main strategy for limiting COVID-19 spread in the population necessarily relied on non-pharmaceutical interventions of variable effectiveness, including individual measures such as personal protective equipment and social distancing, as well as society-wide restrictions such as lockdowns [1]. Later on, rapid development of vaccines provided a much-needed pharmaceutical approach for curbing COVID-19. Although mass vaccination has resulted in widespread immunity against SARS-CoV-2, some of the key concerns remain, including efficacy against newly emerging variants [2], level of protection in immunocompromised individuals [3], and the logistics of mass vaccination, particularly in lower-income economies [4]. Taken together, these considerations highlight a significant unmet need for development of alternative strategies for mitigating COVID-19.

One potentially promising approach involves repurposing of previously characterised drugs [5–8]. Notwithstanding the early high-profile failures of hydroxychloroquine [9] and ivermectin [10], more recently, it has been shown that antidepressant drugs (AD) may be associated with improved outcomes in COVID-19 patients [11–13]; furthermore, one AD (fluvoxamine) has shown efficacy in preventing severe COVID-19 in clinical trials [14–17], and another (fluoxetine) was associated with a slight decrease in mortality in a large cohort of COVID-19 patients [18]. The efficacy of fluvoxamine in symptomatic COVID-19 patients, however, remains controversial [19, 20], and the general utility of ADs for COVID-19 prevention has not been assessed.

Studies in cell-based models indicate that ADs may target cell biological mechanisms implicated in early stages of SARS-CoV-2 infection, hinting at the potential prophylactic effect of ADs [21–24]. To investigate the potential link between ADs and protection against COVID-19 infection, we present analysis of association between positive COVID-19 test result incidence and prior AD exposure in a cohort of community-dwelling mental health outpatients.

## Methods

### Study design, data source, and population

We conducted an observational, retrospective, matched cohort study of individuals admitted to the 4 inpatient care units (Bethlem Royal Hospital, Lambeth Hospital, Lewisham Hospital, and Maudsley Hospital) affiliated with the South London and Maudsley (SLaM) NHS Foundation Trust during the 1st wave of the COVID-19 pandemic of 2020. SLaM provides near-monopoly comprehensive mental health services to a geographic catchment of 1.3 million residents in four boroughs of south London. SLaM has used electronic health records across all its services since 2006 and its Clinical Record Interactive Search (CRIS) platform was set up in 2008 to provide researcher access to de-identified data from these records within a robust governance infrastructure. CRIS has been subsequently developed through a range of data linkages and natural language processing (NLP) algorithms [25], and the platform has provided data for over 250 peer-reviewed publications to date. Using CRIS, we extracted data on admissions of patients aged 18 years or older to SLaM inpatient facilities between 1 April and 31 December 2020. PCR or antigen tests for COVID-19 were routinely performed at admission and during the inpatient stay over that period. The criterion for inclusion in this study was the conclusive positive or negative COVID-19 test result(s) during inpatient stay in a SLaM inpatient unit. Characteristics of the study cohort are presented in Table 1.

**Table 1:** Characteristics of the study cohort

***Characteristics***	***COVID-19 Negative, n= 5,462 (%)***	***COVID-19 Positive, n= 202 (%)***	***Test statistics***
Gender*			0.031^£^, 1, 0.89
Female	2,506 (46.1)	92 (45.5)	
Male	2,935 (53.9)	110 (54.5)	
*Missing data (% of total patients)*	*21(0.4)*	*0 (0.0)*	
Ethnicity*			0.887^£^, 1, 0.35
Non-white	3,052 (58.9)	119 (62.3)	
White	2,127 (41.1)	72 (37.7)	
*Missing data (% of total patients)*	*283 (5.2)*	*11 (5.4)*	
Age group (no missing data)*			19.601^£^, 6,<0.001
18-29	1,595 (29.2)	39 (19.3)	
30- 39	1,342 (24.6)	59 (29.2)	
40- 49	973 (17.8)	25 (12.4)	
50- 59	844 (15.5)	41 (20.3)	
60- 69	424 (7.8)	18 (8.9)	
70- 79	215 (3.9)	13 (6.4)	
80 & over	69 (1.3)	7 (3.5)	
Primary mental health diagnosis (ICD-10) at index date*			
F01-F09: Mental disorders due to known physiological conditions	120 (2.2)	14 (6.9)	4.340^$^, <0.001
F10-F19: Mental and behavioural disorders due to psychoactive substance use	240 (4.4)	6 (3.0)	0.975^$^, 0.33
F20 -F29: Schizophrenia, schizotypal, delusional, and other non-mood psychotic disorders	2,538 (51.1)	104 (56.5)	1.404^$^, 0.16
F30-F39: Mood [affective] disorders	1,071 (21.6)	28 (15.2)	2.029^$^, 0.04
F40-F48: Anxiety, dissociative, stress-related, somatoform and other nonpsychotic mental disorders	241 (4.9)	5 (2.7)	1.326^$^, 0.18
F50-F59: Behavioural syndromes associated with physiological disturbances and physical factors	99 (2.0)	3 (1.6)	0.344, 0.73
F60-F69: Disorders of adult personality and behaviour	474 (9.5)	19 (10.3)	0.361^$^, 0.719
F70-F79: Intellectual disabilities	16 (0.3)	0 (0.0)	0.771^$^, 0.44
F80-F89: Pervasive and specific developmental disorders	44 (0.9)	0 (0.0)	1.281^$^, 0.21
F90-F98: Behavioural and emotional disorders with onset usually occurring in childhood and adolescence	12 (0.2)	0 (0.0)	0.667^$^, 0.50
Z00.4 - General psychiatric examination, not elsewhere classified	109 (2.2)	5 (2.7)	0.477^$^, 0.63
*Missing primary diagnosis(% of total patients)*	*498 (9.1)*	*18 (8.9)*	
HoNOS subscale scores >1*			
Agitation problems	2206 (44.2)	117 (61.3)	4.650^$^, <0.001
Self-injury problems	1006 (20.2)	24 (12.6)	3.150^$^, <0.001
Drinking and substance misuse problems	1710 (34.3)	60 (31.4)	0.815^$^, 0.42
Cognitive problems	1100 (22.0)	68 (35.6)	4.400^$^, <0.001
Physical illness problems	1218 (24.4)	65 (34.0)	3.025^$^, <0.001
Hallucination problems	2736 (54.8)	131 (68.6)	3.756^$^, <0.001
Depressed problems	2063 (41.3)	65 (34.0)	2.013^$^, 0.05
Relationship problems	1805 (36.2)	67 (35.1)	0.031^$^, 0.76
Daily living problems	1430 (28.7)	74 (38.7)	3.016^$^, <0.001
Living condition problems	1327 (26.6)	57 (29.8)	0.997^$^, 0.32
Occupational problems	1602 (32.1)	73 (38.2)	1.776^$^, 0.08
*Missing data (% of total patients)*	*471 (8.6)*	*11 (5.4) *	
**Type of medication start date mentioned 90 days before index admission date**			
Atypical (not mirtazapine)	39 (0.7)	2 (1.0)	0.452^$^, 0.65
MAOI	8 (0.1)	0 (0.0)	0.544^$^, 0.59
Mirtazapine	518 (9.5)	13 (6.4)	1.456^$^, 0.15
SNRI	225 (4.1)	3 (1.5)	1.870^$^, 0.06
SSRI	1,020 (18.7)	21 (10.4)	2.983^$^, <0.001
TCA	113 (2.1)	4 (2.0)	0.087^$^, 0.93
Any antidepressant	1,513 (27.7)	34 (16.8)	3.404^$^, <0.001

*Percentages calculated excluding missing data

^$^To compare two groups difference in proportion test was used; Z value, *p*-value reported

^£^To compare difference in frequencies chi squared test was used; Chi squared test value, degree of freedom and *p*-value reported

### Exposure and outcome

For exposure, we used a natural language processing (NLP) algorithm to identify the medications mentioned in the patient’s record in a 6-month window before or after referral, which provides a validated proxy measure for drug receipt [26]. The list of medications can be found in Table 2. We established use of the following medication classes: atypical (not mirtazapine), monoamine oxidase inhibitors (MAOI), mirtazapine, serotonin and norepinephrine reuptake inhibitors (SNRI), selective serotonin reuptake inhibitors (SSRI), tricyclic antidepressants (TCA), and any antidepressants mentioned above within 31, 62, or 90 days preceding the index hospital admission.

**Table 2:** Drugs assessed in this study

*Drug Class*	*Drug name*
Selective serotonin reuptake inhibitor (SSRI)	citalopram dapoxetine escitalopram fluoxetine fluvoxamine paroxetine sertraline
Serotonin and norepinephrine reuptake inhibitor (SNRI)	venlafaxine duloxetine reboxetine
Atypical	mirtzaphine vortioxetine bupropion trazodone vilazodone
Tricyclic antidepressant (TCA)	amitriptyline clomipramine imipramine lofepramine nortriptyline trimipramine
Monoamine oxidase inhibitor (MAOI)	tranylcypromine phenelzine isocarboxazid moclobemide

The primary outcome measure was the result of the laboratory test for COVID-19 (antigen or PCR). Any incidence of a positive COVID-19 test result during the inpatient stay was categorised as ‘positive’. For categorisation as ‘negative,’ all COVID test results during inpatient stay were required to be negative.

### Covariates and predictors

We ascertained age at the time of admission, gender, and ethnicity (dichotomised to white and non-white) as recorded at the time of hospital admission. We identified the diagnosis given closest to hospital admission in structured fields. According to the International Statistical Classification of Diseases and Related Health Problems (WHO ICD-10) [27] criteria, we established the following diagnosis groups:

- F01-F09: Mental disorders due to known physiological conditions;
- F10-F19: Mental and behavioural disorders due to psychoactive substance use;
- F20-F29: Schizophrenia, schizotypal, delusional, and other non-mood psychotic disorders;
- F30-F39: Mood [affective] disorders;
- F40-F48: Anxiety, dissociative, stress-related, somatoform and other nonpsychotic mental disorders;
- F50-F59: Behavioural syndromes associated with physiological disturbances and physical factors;
- F60-F69: Disorders of adult personality and behaviour;
- F70-F79: Intellectual disabilities;
- F80-F89: Pervasive and specific developmental disorders;
- F90-F98: Behavioural and emotional disorders with onset usually occurring in childhood and adolescence.

Mental and physical health problems as well as functional difficulties were scored using the Health of the Nation Outcome Scales (HoNOS) [28]. This is a routinely used measure in British mental health services and the most recent scores were extracted at the time of the index admission. Each subscale is rated on a scale ranging from 0 (no problem) to 4 (severe or very severe problem); to simplify interpretation, we dichotomised the scores to ‘minor or no problems’ (scores 0 or 1) and ‘mild to severe problems’ (scores 2 to 4).

### Statistical techniques

Initially, *χ*^2^ tests and *Z*-score statistics were used to analyse COVID-19 test results for each covariate. Logistic regression models were then assembled to quantify odds ratios (ORs) for the associations between antidepressant medication receipt and incidence of COVID-19 positive test result, applying the above sub-categorisation of antidepressants and timing as secondary analyses. 95% confidence intervals (CI) and *P*-values for ORs were calculated, and *P*-values < 0.05 were considered statistically significant. Initially unadjusted logistic regression analyses were carried out, followed by adjustments for sociodemographic factors (gender, age, ethnicity) and then further adjustments for those significant HoNoS subscales and primary mental health diagnosis. Finally, primary analyses (only patients receiving any antidepressant medication) were stratified by primary mental health diagnosis measured using ICD-10 diagnosis at the time of index hospital admission. All statistical analyses were conducted with STATA version15 [29].

## Results

We leveraged data from the CRIS platform, which provides research access to deidentified electronic clinical records for SLaM^18^. During the study period (1 April–31 December 2020), 5664 cases of mental health inpatient care admission at SLaM facilities had been tested for COVID-19, with 202 (3.56%) testing positive. Characteristics of the study cohort are presented in Table 1. By ICD-10 code, the most prevalent primary diagnoses were in the F2 (schizophreniform) category, and the second most common in the F3 (mood disorders) category.

We then queried CRIS for mentions of ADs in the clinical records of patients within the time period of 90 days preceding the date of admission. The list of drugs included in the query is presented in Table 2. 27.7% percent of COVID-19-negative cases had at least one AD mention within 90 days preceding admission, compared to 16.8% of COVID-19-positive cases. Accordingly, the occurrence of a positive COVID-19 test result in patients with an AD mention was significantly lower than in those without (2.2 vs 4.1%, *p* = 0.000663, *χ*^2^ test). Most prescribed ADs belonged to the SSRI class, in line with their prevalence in treatment of major depressive disorder [30]: two thirds (67%) of the cases with AD mention within 90 days (Table 1), and the occurrence of a COVID-19-positive test result in patients with a recent SSRI record was significantly less than in those without (2.0 vs 3.9%, *p* = 0.002853, *χ*^2^ test). Associations with other AD classes were not statistically significant.

**Fig. 1﻿:**
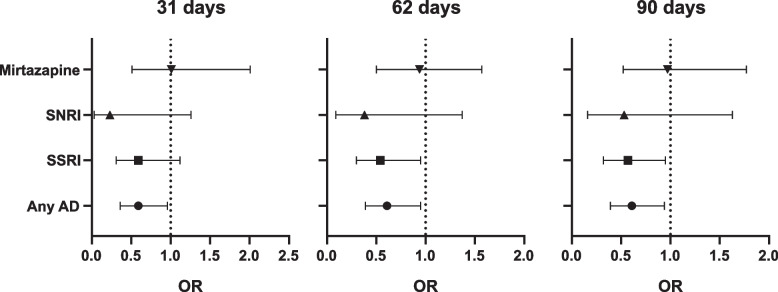
Adjusted odds ratios (OR) at 31, 62, and 90 days (related to Table 3). Error bars correspond to 95% CI

To further investigate the relationship between AD/SSRI and COVID-19 test results, we performed multiple logistic regression analysis (Table 3). We found significant adjusted associations for AD receipt within 31, 60, and 90 days before admission and incidence of positive COVID-19 test results. Similar associations were found for SSRI receipt within 62 and 90 days prior to admission in adjusted models (Fig. 1). Following stratification by diagnosis (Table 4), associations were similar in direction across all groups apart from substance use disorders and were strongest in organic disorders (F0), mood disorders (F3), and anxiety disorders (F4).

**Table 3:** Strength of the association between any antidepressants medication receiving status and COVID-19 positive test result at when medication was mentioned 31 days, 62 days and 90 days for the first time before index hospital admission [Odds ratios (95% CI), P value]. Statistically significant association in bold

***Type of*** ***medicati*** ***on***	***Adjustments***	***31 days***	***62 days***	***90 days***
Any drug received	Unadjusted	**0.52 (0.34, 0.81),** **<0.001**	**0.51 (0.35, 0.76),** **<0.001**	**0.53 (0.36, 0.77),** **<0.001**
	Adjusted for sociodemographic factors (gender, age, ethnicity)	**0.55 (0.35, 0.86),** **0.01**	**0.52 (0.35, 0.79),** **<0.001**	**0.54 (0.37, 0.80),** **<0.001**
	^a^Further adjusted for HoNoS symptoms and primary mental health diagnosis)	**0.59 (0.36, 0.96),** **0.03**	**0.61 (0.39, 0.95),** **0.03**	**0.61 (0.39, 0.94),** **0.02**
SSRIs	Unadjusted	**0.52 (0.30, 0.90),** **0.02**	**0.50 (0.31, 0.8),** **<0.001**	**0.51 (0.32, 0.80),** **<0.001**
	Adjusted for sociodemographic factors (gender, age, ethnicity)	0.59 (0.34, 1.03), 0.06	**0.53 (0.32, 0.87),** **0.01**	**0.55 (0.34, 0.88),** **0.01**
	^a^Further adjusted for HoNoS symptoms and primary mental health diagnosis)	0.59 (0.31, 1.12), 0.10	**0.54 (0.30, 0.95),** **0.03**	**0.57 (0.32, 0.95),** **0.03**
SNRIs	Unadjusted	0.16 (0.02, 1.14), 0.07	0.25 (0.06, 1.02), 0.05	0.35 (0.11, 1.11), 0.07
	Adjusted for sociodemographic factors (gender, age, ethnicity)	0.16 (0.02, 1.12), 0.07	0.25 (0.06, 1.02), 0.05	0.35 (0.11, 1.11), 0.08
	^a^Further adjusted for HoNoS symptoms and primary mental health diagnosis)	0.23 (0.03, 1.66), 0.15	0.38 (0.09, 1.37), 0.10	0.53 (0.16, 1.63), 0.24
Mirtazapine	Unadjusted	0.73 (0.38, 1.39), 0.34	0.65 (0.36, 1.18), 0.16	0.66 (0.37, 1.16), 0.15
	Adjusted for sociodemographic factors (gender, age, ethnicity)	0.71 (0.37, 1.38), 0.32	0.64 (0.35, 1.16), 0.14	0.65 (0.36, 1.15), 0.14
	^a^Further adjusted for HoNoS symptoms and primary mental health diagnosis)	1.01 (0.51, 2.01), 0.97	0.94 (0.50, 1.57), 0.64	0.97 (0.52, 1.77), 0.91

[Odds ratios (95% CI), *P* value]. Statistically significant association in bold

^a^Adjusted for all significant HoNoS subscale and primary mental health diagnosis variables in the Table 1 (F01-F09: Mental disorders due to known physiological conditions, F30-F39: Mood [affective] disorders, Agitation problems, Self-injury problems, Cognitive problems, Physical illness problems, Hallucination problems, Depressed problems, Daily living Problems

**Table 4:** Association between any antidepressant medication receiving status and COVID-19 positive test result stratified by primary mental health condition at the time of index hospital admission^*^ [Odds ratios (95% CI), P value]. Statistically significant association in bold

***Primary diagnosis of the index mental health condition***	***31 days***	***62 days***	***90 days***
F01-F09: Mental disorders due to known physiological conditions	0.27 (0.03, 2.87), 0.28	0.17 (0.01, 2.09), 0.17	0.16 (0.01, 1.97), 0.15
F10-F19: Mental and behavioural disorders due to psychoactive substance use	**20.33 (1.59, 26.20), 0.02**	**7.82 (1.01, 22.27), 0.05**	**15.02 (1.09, 28.05), 0.04**
F20-F29: Schizophrenia, schizotypal, delusional, and other non-mood psychotic disorders	0.54 (0.21, 1.35), 0.19	0.75 (0.37, 1.53), 0.43	0.69 (0.34, 1.40), 0.31
F30-F39: Mood [affective] disorders	**0.25 (0.05, 0.94), 0.04**	**0.28 (0.08, 0.98), 0.04**	**0.37 (0.13, 0.97), 0.04**
F40-F48: Anxiety, dissociative, stress-related, somatoform and other nonpsychotic mental disorders	0.18 (0.06, 1.59), 0.17	0.16 (0.04, 1.72), 0.15	0.19 (0.02, 2.02), 0.17
F50-F59: Behavioural syndromes associated with physiological disturbances and physical factors	0.72 (0.20, 16.08), 0.49	0.78 (0.25, 17, 09), 0.84	0.84 (0.23, 23.09), 0.94
F60-F69: Disorders of adult personality and behaviour	1.86 (0.48, 7.30), 0.37	1.86 (0.44, 7.84), 0.39	1.21 (0.29, 4.96), 0.78

^*^Adjusted for age, gender, ethnicity and all significant HoNoS subscale and primary mental health diagnosis variables in the table 1 (F01-F09:  Mental disorders due to known physiological conditions, F30-F39:  Mood [affective] disorders, Agitation problems, Self-injury problems, Cognitive problems, Physical illness problems, Hallucination problems, Depressed problems, Daily living problems

## Discussion

The results of this study indicate that prior receipt of ADs and specifically SSRIs in community-dwelling mental health outpatients was associated with a decreased likelihood of COVID-19 incidence, suggesting that these drugs may have a protective effect against COVID-19 in this population. A key strength of this study is the large number of participants. Furthermore, the focus on the 1st wave of COVID-19 avoids the confounding effects of the ‘herd immunity’ in the population due to mass vaccination and/or previous exposure to COVID-19 in the following time period [31]. Importantly, exposure to both ADs and COVID-19 occurred prior to hospitalisation, reflecting the ‘real-life’ situational value of the study, which is further enhanced by the diverse socio-economical composition of the study cohort (Table 1). Our findings are consistent with decreased COVID-19 incidence in middle-aged and older adults with self-reported history of psychotropic drug use [32]. Interestingly, decreased COVID-19 incidence has also been reported for mental health inpatients using anti-psychotic medications [33], further underscoring the link between psychotropic drugs and protection against COVID-19.

SSRI treatment is associated with a dropout rate of 28% over the standard 6-month treatment course [34]. Given the study design, it can be expected that a proportion of cases did not adhere to the prescription regimen, suggesting that the observed effect may be an underestimation. Nevertheless, the effect size reported here is considerably larger than 8% reduction in overall mortality associated with record of SSRI in a large cohort of COVID-19 cases [18], being more similar to the 36% reduction by fluvoxamine in risk of hospitalisation for outpatients with COVID-19 [17] and to the 44% reduction in risk of intubation or death for hospitalised patients with COVID-19 [11]. In the context of the above studies, our findings hint that ADs/SSRIs may be at least as effective in preventing COVID-19 as in treating it, providing impetus for further investigation of their clinical utility in the general population.

The mechanisms underlying the putative protective effects of SSRIs in COVID-19 remain unclear. Some of the proposed cell biology mechanisms include blockade of viral replication [21, 23], modulation of endocytic trafficking [6, 22], phospholipidosis [35], and anti-inflammatory action through inhibition of cytokine release [36, 37]. In turn, candidate molecular targets for SSRI action include acid sphingomyelinase [12, 24], sigma receptor [38], and even the lipid bilayer of the cell membrane itself [39]. Further mechanistic insight into the role of SSRIs outside the central nervous system will require detailed investigation of physiology and cell biology of SSRIs in appropriate experimental systems, with particular consideration given to pharmacokinetics of therapeutically relevant SSRI concentrations.

### Study limitations

The study has a number of important limitations, largely due to its retrospective nature and focus on a cohort of mental health patients. It cannot demonstrate a causal relationship between medications and COVID-19 test results; indeed, the protective effect is consistent with either decreased infection rate or increased recovery rate from COVID-19.

The time period of the study was limited to the first wave of the COVID-19 pandemic, which presented novel adverse effects on mental health [40]; also, during this period, COVID-19 was associated with the original strain of SARS-CoV-2 rather than its subsequently documented variants. Owing to the numbers of participants and prevalence of multiple drug prescriptions, it was not possible to investigate the association between COVID-19 and individual drugs; compared to SSRIs, the number of participants receiving other drug classes was low (Table 1), and it was also not possible to assess older-generation drugs, including fluvoxamine. The study did not account for dosage regimen; also, it was not possible to determine medication adherence from the available data.

Finally, although the association of interest appeared to be present across a range of diagnostic groups, the possibility cannot be ruled out that antidepressant use may have been a marker of personal or behavioural factors conferring protection, e.g. compliance with societal restrictions and/or personal protection measures in place at the time. To address the above limitations and to further corroborate the findings of this study, randomised prospective clinical studies for selected ADs in the general population will be of essence.

## Conclusions

Our results suggest that ADs, and SSRIs in particular, may provide a degree of protection against COVID-19, specifically SARS-CoV-2 infection. This evidence lends some support to further investigation of drug repurposing as an complementary strategy to vaccination in appropriate contexts, especially considering the key advantages of ADs viz. well-characterised safety profile, low price, and ready availability. Identification of affordable and safe drugs that reduce the risk of COVID-19 is likely to be relevant for the global pandemic response, particularly in cases and situations where mass vaccination may be problematic.

In the longer term, there may be merit in investigating the utility of ADs/SSRIs for treatment of other respiratory infections using similar cell biological mechanisms to COVID-19, e.g. influenza [41]. For now, one can hope that the results from this study will contribute to the public health policy debate on COVID-19 management, help re-establish drug repurposing in the context of COVID-19 treatment and highlight the potential for wider clinical benefits of psychotropic drugs.

## Data Availability

All data generated or analysed during this study are included in this published article (Tables 1, 2, 3, and 4).

## Abbreviations

AD: Antidepressant
CI: Confidence interval
COVID-19: Coronavirus disease 2019
CRIS: Clinical Record Interactive Search
HoNOS: Health of the Nation Outcome Scale
ICD-10: International Statistical Classification of Diseases and Related Health Problems (ICD), 10^th^ revision
MAOI: Monoamine oxidase inhibitor
NHS: National Health Service﻿
NIHR: National Institute for Health and Care Research
NLP: Natural language processing
OR: Odds ratio
PCR: Polymerase chain reaction
SNRI: Serotonin and norepinephrine reuptake inhibitor
SSRI: Selective serotonin reuptake inhibitor
SLaM: South London and Maudsley
TCA: Tricyclic antidepressant
WHO: World Health Organization
